# Thrombotic Complications Associated with Immune Checkpoint Inhibitors

**DOI:** 10.3390/cancers13184606

**Published:** 2021-09-14

**Authors:** Tzu-Fei Wang, Alok A. Khorana, Marc Carrier

**Affiliations:** 1 Department of Medicine, University of Ottawa, The Ottawa Hospital and Ottawa Hospital Research Institute, Ottawa, ON K1H 8L6, Canada; mcarrier@toh.ca; 2 Taussig Cancer Institute, Cleveland Clinic and Case Comprehensive Cancer Center, Cleveland, OH 44106, USA; khorana@ccf.org

**Keywords:** venous thromboembolism, arterial thrombosis, cancer-associated thrombosis, immune checkpoint inhibitors, anticoagulation

## Abstract

**Simple Summary:**

Patients with cancer commonly develop blood clots, which can cause issues including hospitalizations and complications and can affect cancer treatments. Cancer therapies can be one of the reasons for blood clots. A type of cancer therapy called “immune checkpoint inhibitors” has been used more and more often in recent years for different types of cancer. Recent reports revealed an increasing concern of blood clots related to immune checkpoint inhibitors. In this review, we will summarize data from the available studies and discuss the rates, risk factors, prevention, and treatment strategies for blood clots related to immune checkpoint inhibitors.

**Abstract:**

Thromboembolism is a common complication in patients with cancer and is associated with significant morbidity and mortality. Anticancer treatment is a known risk factor of cancer-associated thrombosis. Immune checkpoint inhibitors have become a mainstay of treatment in various cancers. Both venous and arterial thrombosis have been increasingly reported as adverse events associated with immune checkpoint inhibitors in recent studies, with a cumulative incidence of venous thrombosis to be 5–8% at 6 months and over 10% at 12 months. Additionally, rates of approximately 1–5% for arterial thrombosis were reported at 12 months. Data also showed an association of thromboembolism with adverse survival. Many pertinent clinical questions in this population deserve further investigation, including the risks of thrombosis associated with immune checkpoint inhibitors as compared to those with traditional systemic therapy, associated risk factors, and the optimal prevention and treatment strategies. In this review, we synthesize data from available literature, provide relevant information for clinicians and potential future directions for research.

## 1. Introduction

Patients with cancer have a 12-fold increased risk of venous thromboembolism (VTE) compared to the general population, with the risk further increased to 23-fold in patients receiving chemotherapy or targeted therapy [1]. Cancer-associated thrombosis is linked with significant morbidity that could lead to hospitalizations, delay in cancer treatment, and even mortality. As a result, thromboembolism is the second leading cause of death in cancer patients [2]. Many factors can contribute to the increased risk of thrombosis in patients with cancer, including tumor-related factors such as type and stage of malignancy; patient-related factors such as age, history of VTE, obesity, or other co-morbidities; and treatment-related factors such as surgery or systemic anticancer therapies [3].

In recent years, immune checkpoint inhibitors (ICIs) have revolutionized the landscape of oncology treatment. ICIs are monoclonal antibodies that target proteins that negatively regulate the immune system called immune checkpoints, including programmed cell death (PD)-1 and cytotoxic T lymphocyte-associated antigen 4 (CTLA-4) [4]. PD-1 and CTLA-4 are typically expressed on T cells and bind to PD-1 ligands on tumor cells and CD80/CD86 on antigen-presenting cells, respectively. This leads to T cell inactivation to keep the immune response in check [4]. However, tumor cells also commonly utilize this pathway to escape the immune system. Blocking these pathways results in the activation of T cells to target and kill tumor cells [4]. The United States Food and Drug Administration (FDA) approved the first ICI in 2011; by 2020, seven ICIs had been approved by the FDA in at least 15 cancers, and patients eligible for ICIs significantly increased from 1.5% in 2011 to 43.6% in 2018 [5,6]. They have become a mainstay of treatment and are widely used in various cancers, including non-small cell lung cancer (NSCLC), melanoma, renal cell carcinoma, head and neck cancer, and more. Available ICIs target PD-1 (nivolumab, pembrolizumab, cemiplimab) or its ligands (atezolizumab, avelumab, durvalumab) as well as CTLA-4 (ipilimumab). ICIs are known to be associated with a wide range of immune-related toxicities, including gastrointestinal, skin, thyroid, or hematological findings such as autoimmune hemolytic anemia or thrombocytopenia. 

Thrombosis had not been a commonly reported adverse event in initial phase III clinical trials leading to the approval of ICIs. However, after the routine clinical use of these agents, multiple studies started to report concerns for thrombosis, although rates varied widely. Data were also conflicting on whether ICIs were indeed associated with a higher risk of thrombosis than traditional chemotherapy.

Therefore, we review the current literature on venous or arterial thrombosis associated with ICIs. We will address potential mechanisms of thrombosis, summarize the reported rates of venous and arterial thrombosis among various studies, discuss treatment and prevention of thrombotic complications and risk factors for thrombosis in patients receiving ICIs. 

## 2. Methods

For this review, we performed a comprehensive literature search of the MEDLINE database from inception to 11 July 2021. Search terms included (“thromboembolism” OR “thrombosis”) AND (“cancer” OR “malignancy”) AND (“immune checkpoint inhibitor” OR “immunotherapy”). All prospective or retrospective cohort studies, meta-analyses, and review articles pertinent to the topic were reviewed. After excluding duplicates, 690 articles were screened by titles and abstracts. The references in the relevant articles were also manually searched for related studies. Overall, 18 retrospective studies reporting incidences and/or outcomes of venous and/or arterial thrombosis in patients on ICIs [1,7,8,9,10,11,12,13,14,15,16,17,18,19,20,21,22,23] and 4 additional relevant systematic reviews and meta-analyses [24,25,26,27] were identified and included in this review. 

## 3. Mechanism of Thrombosis Related to Immune Checkpoint Inhibitors

While the exact mechanisms of thrombosis associated with ICIs remain to be elucidated, several pathways have been considered. Immune checkpoint blockage has been demonstrated to be associated with a pro-inflammatory state and elevated levels of inflammatory cytokines [28]. Furthermore, mouse models showed that PD-1 played a crucial role in downregulating pro-atherogenic T cells, and blockage of PD-1 could accelerate atherogenesis with increased infiltration of macrophages and pro-inflammatory T cells in atherosclerotic plaques and enhance vascular inflammation and atherosclerosis [29,30]. The promotion of atherosclerotic plagues after ICI use was demonstrated in pre-clinical animal models and could potentially contribute to the increased arterial thrombotic events [31]. 

In addition, activated T cells can induce synthesis of tissue factor in monocytes and macrophages [32] and is hypothesized to be one of the mechanisms that promote hypercoagulability [33]. To further delineate the pathogenesis of ICI-associated VTE, Roopkumar et al. analyzed pre-ICI blood samples from 15 individuals who subsequently developed VTE on ICIs compared to 10 without VTE on ICIs [21]. In patients who developed a VTE, they found a significant increase in the numbers of total myeloid-derived suppressor cells (MDSCs) and elevated levels of inflammatory biomarkers, including CXCL8 (chemokine ligand), soluble vascular cell adhesion molecule 1, and clustering of other inflammatory cytokines including IL-1β, IL-6, TNF prior to ICI. The potential link between MDSCs and thrombosis is intriguing and could be related to platelet activation triggered by MDSCs, as found in an in-vitro study when MDSCs were exposed to metastatic tumor cells [34], as well as in other thromboinflammatory disorders such as the recent severe acute respiratory syndrome coronavirus 2 (SARS-CoV-1) infection [35]. MDSCs can also release neutrophil extracellular traps (NETs) induced by CXCL8-involved pathways and contribute to a heightened risk of thrombosis [21,36]. Moreover, the elevation of other cytokines is hypothesized to cause activation of endothelium and platelets and activate the pathologic process of immunothrombosis [21,37]. These inflammatory biomarkers could potentially be used to identify patients at high risk for VTE on ICIs in the future. 

## 4. Incidence of Thrombosis 

### 4.1. Venous Thromboembolism (VTE)

A systematic review and meta-analysis including 68 studies (18 retrospective studies with the rest being clinical trials investigating the efficacy of ICIs, N = 20,273) showed an incidence of VTE and ATE of 2.7% (95% CI 1.4–5.4%) and 1.1% (95% CI 0.65–1.45%) in cancer patients receiving ICIs, respectively [26]. The authors concluded that ICIs were associated with low incidences of thromboembolic events, not different from chemotherapy alone. However, this systematic review was limited in that the definition of thromboembolic events and the follow-up duration in each study varied and were often unclear. In addition, 40% of the eligible studies did not report on thromboembolic events and were excluded from the analysis [38]. In contrast to the low rates reported from initial clinical trials, more recent studies revealed a much higher rate of thrombosis. 

Table 1 and Table 2 summarize the characteristics and reported outcomes in studies focusing on VTE and/or ATE in patients receiving ICIs. A total of 18 studies were included; all were retrospective, including two population cohorts. There was a male predominance, with the majority of patients having metastatic disease (80–100%). The most common cancer types included lung, renal cell, and melanoma (Table 1). The follow-up duration varied widely, ranging from 6 to 37.8 months, and accordingly, the incidence of thrombosis also varied, and it was difficult to conclude a definitive rate. In general, the cumulative incidence of VTE is approximately 5–8% at 6 months and over 10% at 12 months (Table 2). These rates are much higher than the reported rate of 2.7% from the previous meta-analysis. These findings suggest concerning evidence that thromboembolic events are commonly under-reported or under-estimated in oncology clinical trials where the primary goal is to evaluate the effectiveness of anticancer therapies, and thromboembolism is often reported as adverse events (not primary or secondary outcomes) by using Common Terminology Criteria for Adverse Events (CTCAE). The under-reporting of thromboembolic complications has also been reported in colorectal and pancreatic cancer trials [39,40]. As such, the subcommittee on Haemostasis and Malignancy of the International Society on Thrombosis and Haemostasis (ISTH) has called for standardization of reporting and analysis of VTE events in patients with cancer [41]. This is important as inconsistent reporting could lead to erroneous estimates of true event rates and missing potentially important adverse events. 

Additionally, whether ICIs are associated with an increased risk of VTE compared to traditional chemotherapy is unclear, as patients receiving ICIs and chemotherapy are usually not directly comparable, and many studies only included patients on ICIs or ICI combinations. Four studies have attempted to compare rates of thrombotic complications in patients receiving ICIs to those receiving chemotherapy. Bar et al. did not find a difference in thrombotic complications in patients on ICIs, chemotherapy alone, or a combination of ICIs and chemotherapy [10]. Similarly, a Danish population cohort study showed a 6-month cumulative incidence of VTE of 4.1% (95% CI 2.3–6.7%) in patients receiving ICIs, comparable to 3.5% (95% CI 3.4–3.6%) found in those receiving chemotherapy [1]. In another study, Icht et al. included 345 patients with NSCLC and reported a 6-month cumulative incidence of VTE of 7.1% in the chemotherapy cohort, compared with 4.5% in the ICI cohort (HR 1.6, 95% CI 0.6–3.9) [17]. However, as expected, the two cohorts were significantly different in baseline characteristics, including more metastatic disease and much less use as first-line therapy in the ICI cohort, which could have affected the results. Lastly, Madison et al. showed that ICIs were associated with a higher risk of thrombosis compared to chemotherapy alone (10.2% vs. 7.6%, respectively) in the univariable analysis, but multivariable analysis failed to show ICI as an independent risk factor for VTE [19], indicating the presence of other confounders. In a report from the FDA Adverse Event Reporting System (FAERS), 1855 patients with ICI-associated thromboembolic events were reported between 2004 and 2019 [42]. Compared to the whole database (which included chemotherapy and protein kinase inhibitors), ICIs were associated with an increased risk of VTE and ATE (reporting odds ratio 2.81 and 1.44, respectively) [42]. However, reporting was voluntary, so biased reporting was highly likely. Therefore, the available data so far did not show a clear differentiation in the rates of thromboembolic events associated with ICIs compared to chemotherapy, although the quality of data remains poor. 

Another important consideration is the prolonged use of ICIs and that the occurrence of thrombosis could spread throughout the duration of use. A recent Danish population-based cohort study of 499,092 patients with first cancer diagnosis between 1997 and 2017 showed that in 370 patients treated with ICIs, a 6-month cumulative incidence of VTE following cancer diagnosis was 4.1% (95% CI 2.3–6.7%), with a 12-month cumulative incidence of 7.1% (95% CI 4.2–11.1%) [1]. This indicated that the heightened risks associated with ICIs continued beyond the initial 6 months. Another analysis of the prospective APOLLO cohort in patients receiving ICIs for NSCLC also showed that two-thirds of the VTE occurred after 6 months [11]. This is in contrast with chemotherapy-related VTE, for which the majority of events occur within the first 6 months of initiating therapy [43]. Furthermore, given their improved effectiveness, patients receiving ICIs have prolonged survival with ongoing treatment, which increases the duration of exposure to ICIs and associated risk for thrombosis. Indeed, APOLLO cohort analysis showed that patients with either venous or arterial thromboembolism had significantly longer treatment durations, receiving a median of 20 cycles compared to 6 in those without thrombotic events [11]. 

Overall, more recent studies have revealed concerns that the risks of ICI-associated VTE were not as low as previously reported in clinical trials focusing on the efficacy of ICIs. However, the actual incidence rates remain to be elucidated. For future trials in cancer patients, it is crucial to standardize reporting of VTE to be objectively confirmed and include important characteristics of VTE events (such as distal vs. proximal, incidental vs. symptomatic, deep vein thrombosis vs. pulmonary embolism, etc.) as well as the standardized duration of follow-up [41].

### 4.2. Arterial Thrombosis

As noted previously, pre-clinical studies have shown that blockage of PD-1 or CTLA-4 could enhance atherosclerotic inflammation and promote atherosclerosis plaques [29,30]. Four meta-analyses (mostly of clinical trials) have shown a rate of myocardial infraction of ≤ 1% and that of stroke of 1–2% (Table 3), but as with VTE, the rates could be under-reported [24,25,26,27]. Fewer retrospective studies have reported variable rates of ATE, ranging from 1 to 5% at 12 months (Table 2). 

Drobni et al. showed that ICIs were associated with an increased risk of arterial thrombosis compared to other anticancer therapy [13]. Findings were similar when patients were used as their own control to compare the risks of arterial thrombosis before and after receiving ICIs [13]. Interestingly, this was also the only study investigating the use of steroids or statins, commonly used to modulate autoimmune dysfunction or to modify cardiovascular risk factors, respectively, and found benefits with both to attenuate atherosclerotic plaque progression in patients receiving ICIs [13]. Optimization and control of cardiovascular risk factors may provide benefits in this population, which requires further investigation. 

## 5. Treatment of Thrombosis 

Treatment of VTE in patients with cancer on ICIs is not different from other cancer-associated thromboses, which has been covered extensively by recent guidelines and review articles [44,45,46]. Direct oral anticoagulants (DOACs) and low-molecular-weight-heparin are currently the main treatment options for cancer-associated thrombosis. Choice of anticoagulants would depend on tumor types (with the associated risk of bleeding complications), additional risk factors (such as thrombocytopenia, the presence of intracranial tumors, etc.), potential drug-drug interactions (DDI), and patient perspectives [44]. Fortunately, since ICIs are monoclonal antibodies, no significant DDIs with anticoagulants are expected or reported to date. However, ICIs may be used concurrently with other anticancer therapies or supportive care medications, which could still have potential DDIs, and therefore evaluation of DDIs among all concomitant medications remains advisable. 

The optimal treatment of arterial thrombosis in patients with cancer is less clear as data are even scanter. Standard-of-care similar to what is provided for the non-cancer population is typically employed, including the utilization of antiplatelet agents with or without anticoagulation, modification of cardiovascular risk factors such as control of blood pressure, diabetes, smoking cessation, etc., and/or revascularization when indicated. 

## 6. Prevention of Thrombosis

Two recent large RCTs established the role of DOACs as primary thromboprophylaxis in ambulatory cancer patients with a Khorana score ≥ 2 [47,48]. The Khorana score was derived in a patient population starting a new line of chemotherapy, and whether it can be applied to patients on ICIs is not fully understood. Most studies that investigated the Khorana score for its prediction performance in patients receiving ICIs have not found it to be predictive [11,16,17,18,20]. This indicates that patients on ICIs likely have different sets of risk factors compared to those on chemotherapy, and a specific risk assessment model in this population may be needed. 

Many studies have attempted to identify risk factors for thromboembolic events in this population (Table 2). However, most studies were small and single-center, and therefore, not surprisingly, different sets of risk factors were identified from each study, including a history of thromboembolic disease, metastasis, poor performance status, lung cancer, or melanoma, and more (Table 2). It is worth noting that many of these studies tended to perform multivariable analyses with many variables from datasets with a limited number of patients and associated outcome events, leading to potential model overfit that could make results questionable [10,12,15,16,18]. Future research is needed to derive and validate a specific risk assessment model in a larger patient population receiving ICIs, which can help risk prediction and tailor thromboprophylaxis, if needed, in this growing population. 

For primary prevention of arterial events, standard-of-care as in non-cancer population can be of benefit, including careful assessment of cardiovascular risk factors, such as smoking, obesity, hypertension, hyperlipidemia, diabetes, etc., and aggressive modification of these risk factors with lifestyle change or medications such as statins. As noted previously in the study from Drobni et al., steroids or statins may modify atherosclerotic plaques while on ICIs, but more research is needed [13]. In addition, aspirin or statin use at the time of ICI initiation was shown to be associated with an increased response rate to ICIs in a multicenter retrospective study of 1012 patients. This finding is interesting and hypothesis-generating for future investigation [49]. 

## 7. Survival and Thrombosis in Patients Receiving Immune Checkpoint Inhibitors

In patients receiving ICIs, several studies have shown that the occurrence of thrombosis was associated with worsening survival [10,11,20,21,22] while others have not [14,15,16] (Table 2). Possible explanations of worse survival in patients who developed VTE include that thrombosis is an indicator for more advanced cancer stage, worse prognosis, poorer performance status, and/or thrombosis or anticoagulation-related mortality. 

Pre-clinical studies have demonstrated that coagulation factors such as factor X can help tumors escape the immune system [50]. Factor X inhibitors such as rivaroxaban were shown to enhance the effects of ICIs and immunity against tumor cells and inhibit tumor growth in mice models [50,51]. It is of great interest whether this finding can be translated into a clinical setting. However, a recent retrospective study showed no difference in response rates, progression-free or overall survival in patients receiving ICIs and therapeutic anticoagulation compared to patients who were not on anticoagulation [52]. Of note, anticoagulation included in this study was not limited to factor X inhibitors (including dabigatran, rivaroxaban, apixaban, enoxaparin, and warfarin). Moreover, the cohort of patients on anticoagulation in the study was significantly older, with poorer performance status, and a higher percentage had lung cancer, all of which were poor prognostic factors [52]. The authors performed a multivariable analysis to adjust for confounders and reached the same conclusions, but residual confounding could be present. More studies with larger sample sizes are needed. 

## 8. Conclusions

ICIs have become the main treatment strategy in cancer and result in prolonged survival. Recent observational studies have shown concerns for increased risks of both venous and arterial thromboses in patients receiving ICIs than previously perceived, and patients with thromboembolic complications while on ICIs had also been shown to have worsened survival in some studies, but whether the risks are higher compared to those associated with chemotherapy remain unclear. It is important for clinicians to be aware of the potential thrombotic complications, to educate patients and recognize signs and symptoms of thrombosis, to allow prompt treatment if needed and avoid complications. Future research to evaluate risk factors and develop robust risk assessment models to allow risk stratification and effective utilization of thromboprophylaxis in this population is needed. Furthermore, whether the concurrent use of anti-Xa inhibitors, aspirin, or statins can enhance the effects of ICIs and lead to better antitumor effects and survival is of great interest. 

## Figures and Tables

**Table 1:** Baseline characteristics of studies including patients on immune checkpoint inhibitors.

Study	Study Design	N	Age (Median, IQR)	Male, % (*n*)	Stage IV, % (*n*)	Type of Cancer
Hegde et al. 2017 [7] abstract	Retrospective	76	N/A	N/A	N/A	Lung
Ibrahimi et al. 2017 [8] abstract	Retrospective	154	63 (range 23–89)	43% (66)	92% (142)	Lung 20.8% Melanoma 20.1% Ovarian 12.3%
Hsu et al. 2018 [9]	Retrospective	50	58.7 (mean, range 37–80)	58% (29)	74% (37)	All cancers NSCLC 48%
Bar et al. 2019 [10]	Retrospective	1215	52.3% ≥ 65	59% (717)	N/A	All cancers Melanoma 40.5% Lung 28.7%
Nichetti et al. 2019 [11]	Retrospective analysis from prospective APOLLO cohort	217	70 (range 32–90)	62.7% (136)	95.4% (207)	NSCLC
Ando et al. 2020 [12]	Retrospective	122	N/A	74.6% (91)	N/A	Lung, kidney, stomach, urothelial, melanoma
Drobni et al. 2020 [13]	Retrospective (case control and case cross-over)	2842	66 (57–74)	57.4% (1631)	N/A	All cancer NSCLC 28.8% Melanoma 27.9%
Deschˆenes-Simard et al. 2021 [14]	Retrospective	593	66.7 (60.4–72.5)	54.3% (322)	87.2% (368)	NSCLC
Gutierrez-Sainz et al. 2021 [15]	Retrospective	229	64 (range 19–86)	63.8% (146)	96.5% (221)	Lung 48% Melanoma 23.6% RCC 11.8%
Guven et al. 2021 [16]	Retrospective	133	60 (48–66)	64.7% (86)	100% (133)	Renal cell 26.3% Melanoma 24.1%, NSCLC
Icht et al. 2021 [17]	Retrospective	176	66 (60–72)	60.8% (107)	85.8% (151)	NSCLC
Kewan et al. 2021 [18]	Retrospective	552	38.8 (range 26.8–94.8)	65% (359)	100% (552)	All cancers NSCLC 47.3%
Madison et al. 2021 [19]	Retrospective	6127 *	69–71 * (range 30–96)	97.4% (5967)	N/A	Lung
Moik et al. 2021 [20]	Retrospective	672	64 (54–72)	61.3% (412)	85.8% (566)	Melanoma 30.4% NSCLC 24.1% RCC 11%
Mulder et al. 2021 [1]	Population cohort	370	N/A	N/A	N/A	All cancers
Roopkumar et al. 2021 [21]	Retrospective	1686	Mean 64.5 (range 18–93)	60.1% (1014)	90.3% (1523)	Lung 49.6% Melanoma 13.2%
Sussman et al. 2021 [22]	Retrospective	228	65.5 (range 23–91)	67.5% (154)	81.1% (181)	Melanoma
Moik et al. 2021 [23] abstract	Population cohort	3259	N/A	N/A	N/A	All cancers

* including N = 2678 for immune checkpoint inhibitors alone, and N = 3449 for immune checkpoint inhibitors plus chemotherapy, median age reported by groups (immune checkpoint inhibitors alone or immune checkpoint inhibitors plus chemotherapy). Abbreviations: N—number of patients included; SCLC—non-small cell lung cancer; N/A—not available; RCC—renal cell carcinoma.

**Table 2:** Thrombotic outcomes and risk factors in patients on immune checkpoint inhibitors in included studies.

Study	Study Design	N	Follow Up [Median (IQR), Unless Specified]	VTE Incidence (%, 95% CI)	ATE Incidence (%, 95% CI)	Risk Factors for Thrombosis	Comments
Hegde et al. 2017 [7] abstract	Retrospective	76	10.8 mo	18.4	2.6	Female	VTE after ICI did not affect survival, but before ICI did
Ibrahimi et al. 2017 [8] abstract	Retrospective	154	7 mo (198 days)	10.4	0	N/A	VTE was not associated with progression-free survival
Hsu et al. 2018 [9]	Retrospective	50	N/A	2	N/A	N/A	Focused on survival and toxicity No follow up duration nor how VTE assessed
Bar et al. 2019 [10]	Retrospective	1215	12 mo	AVE (including MI, stroke, PE, multisite DVT): 6 mo: 2.6 12 mo: 3.0 AVE plus single site DVT: 6 mo: 4.9 12 mo: 5.8		NSCLC History of AVE Hypertension Dyslipidemia	AVE was associated with worse survival Rate of AVE was similar in ICI vs. chemo vs. ICI+chemo in lung cancer
Nichetti et al. 2019 [11]	Retrospective analysis from prospective APOLLO cohort	217	37.8 mo	7.4	6.5	Current smoker PD-L1 > 50% Not: KS Anticoagulant or antiplatelet agents	TE is associated with worse survival after TE
Ando et al. 2020 [12]	Retrospective	122	N/A Time to thrombosis 90 days (range 6–178)	4.1 Likely 6 mo rate	4.9	History of TE	No follow up duration, unclear definition of TE
Drobni et al. 2020 [13]	Retrospective	2842	2 years	N/A	Composite: 5.35/100 person-yrs MI: 2.49 Stroke: 2.08	Overall study: ICIs, age, h/o stroke, diabetes, hypertension, NSCLC, male, history of radiation	ICI was associated with increased risk of composite cardiovascular events Statin and steroids attenuated atherosclerotic plaque progression
Deschˆenes-Simard et al. 2021 [14]	Retrospective	593	12.7 (4.9–22.7) mo	9.9 (7.5–12.3) 76.5 (59.9–97.8) per 1000 person-years	1.3	Age < 65 Higher PD-L1 level Smoking <12 mo from diagnosis to ICIs	VTE was not correlated with survival
Gutierrez-Sainz et al. 2021 [15]	Retrospective	229	9.8 mo	7 (4–10)	N/A	Female Melanoma	VTE was not an independent factor for shorter survival
Guven et al. 2021 [16]	Retrospective	133	10.1 (5.8–18.5) mo	11.3	N/A	ECOG ≥ 1 Not: KS	Median survival numerically shorter in VTE patients, not significant
Icht et al. 2021 [17]	Retrospective	176	6 mo (187 days)	4.5 (2.1–8.3)	N/A	Not: KS	VTE was associated with death
Kewan et al. 2021 [18]	Retrospective	552	12.1 mo	12.1	1.3	AC use at the time of ICIs (univariable) Not: KS	Median time to VTE 3.8 mo KS predicts overall survival
Madison et al. 2021 [19]	Retrospective	6143 *	6 mo	6.3	2.6	N/A	ICIs were associated with higher risk of thrombosis compared to chemo alone but not significant in multivariable analysis
Moik et al. 2021 [20]	Retrospective	672	8.5 mo	6 mo: 5.0 (3.4–6.9) Overall: 12.9 (8.2–18.5)	6 mo: 1.0 (0.4–2.0) Overall 1.8 (0.7–3.6)	History of VTE Not: KS	VTE (after ICI) was associated with worse survival
Mulder et al. 2021 [1]	Population cohort	370	12 mo	6 mo: 4.1 (2.3–6.7) 12 mo: 7.1 (4.2–11.1)	N/A	N/A	N/A
Roopkumar et al. 2021 [21]	Retrospective	1686	438 days (range 7–1971)	6 mo: 7.1 12 mo: 10.9 Overall: 24	N/A	Younger age Metastasis Biomarkers	VTE was associated with worse survival No difference in VTE incidence with types of ICIs
Sussman et al. 2021 [22]	Retrospective	228	27.3 mo	6 mo: 8.0 (4.9–12.0) 12 mo: 12.9 (8.9–17.7)	6 mo: 2.2 (0.84–4.8) 12 mo: 4.5 (2.3–7.8)	Combination ICI KS ≥ 1 History of CAD Anticoagulation at treatment start	VTE was associated with worse survival
Moik et al. 2021 [23] abstract	Population cohort	3259	24 mo	6 mo: 3.9 (3.3–4.7) 12 mo: 5.7 (4.9–6.6) 24 mo: 7.3 (6.2–8.4)	6 mo: 1.3 (0.9–1.8) 12 mo: 2.2 (1.7–2.8) 24 mo: 3.1 (2.4–3.8)	N/A	Use of ICI was associated with 1.5 to 6.5 fold increased odds of VTE

* including N = 2685 for ICIs alone, and N = 3458 for ICIs plus chemotherapy, median age reported by groups (ICIs alone or ICIs plus chemotherapy). Abbreviations: ATE—arterial thrombosis; AVE—acute vascular events; CAD—coronary artery disease; DVT—deep vein thrombosis; ECOG—Eastern Cooperative Oncology Group; h/o–history of; ICIs—immune checkpoint inhibitors; IQR—interquartile range; KS—Khorana score; MI—myocardial infarction; mo—months; N—number of patients included; N/A—not available; NSCLC—non-small cell lung cancer; PE—pulmonary embolism; TE—thromboembolism; VTE—venous thromboembolism.

**Table 3:** Meta-analyses reporting thrombotic outcomes in patients on immune checkpoint inhibitors.

Study	Search Cut-Off Date	N	Cancer Types	Rate of VTE, % (95% CI)	Rate of ATE, % (95% CI)
Hu et al. [24]	24 March 2017	4828 (22 studies)	NSCLC	Only one PE reported	MI (*n* = 402): 1.0 (0–3.8) Stroke (*n* = 135): 2.0 (0–13.0)
Nso et al. [25]	15 May 2020	4622 (26 studies)	Various	N/A	MI (*n* = 1168): 0.4 (CI 0.1–0.7)
Solina et al. [26]	21 May 2020	20,273 (68 studies)	Various	Overall: 2.7 (1.8–4) PE: 1.6 (0.7–3.2) DVT: 2.7 (1.4–5.4)	Overall: 1.1 (0.5–2.1) MI: 0.7 (0.15–1.15) Stroke: 1.1 (0.65–1.45)
Agostinetto et al. [27]	30 June 2020	35,337 (80 RCTs)	Various	N/A	ICI group: MI: 0.41% (27/6607) Dual ICI: MI: 0.5% (1/202)

Abbreviations: ATE—arterial thrombosis; CI—confidence interval; DVT—deep vein thrombosis; ICI—immune checkpoint inhibitors; MI—myocardial infarction; N—number of patients included; N/A—not available; NSCLC—non-small cell lung cancer; PE—pulmonary embolism; RCTs—randomized controlled trials; VTE—venous thromboembolism.
